# The complete chloroplast genome sequence of *Elsholtzia densa*, a herb with volatile aroma component

**DOI:** 10.1080/23802359.2019.1710597

**Published:** 2020-01-14

**Authors:** Gui Fu, Jing Liu, Junqiao Li

**Affiliations:** a The College of Ecological Environmental and Resources, Qinghai Nationalities University, Xining, China;; b Key Laboratory of Biotechnology Analysis and Test in Qinghai-Tibet Plateau, Qinghai Nationalities University, Xining, China;; c Key Laboratory of Resource Chemistry and Eco-environmental Protection in Tibetan Plateau, Qinghai Nationalities University, State Ethnic Affairs Commission, Xining, China

**Keywords:** *Elsholtzia densa*, chloroplast genome, Labiatae, phylogenetic relationship

## Abstract

The complete chloroplast genome sequence of *Elsholtzia densa* was analysed. The results indicated that the size of the chloroplast genome was 149,095 bp in length with a large single-copy region (LSC) of 81,497 bp, a small single-copy region (SSC) of 17,364 bp, and a pair of inverted repeat (IR) regions of 25,117bp. The overall GC content of the cpDNA genome was 37.86%, while the corresponding values of the LSC, SSC, and IR regions were 35.96%, 31.92%, and 43.16%, respectively. A total of 132 functional genes were identified, including 84 protein-coding genes, 37 tRNA genes, and 8 rRNA genes. The maximum likelihood phylogenetic tree suggested that *E. densa* was closely related to the species in the family Labiatae.

*Elsholtzia densa* belongs to the *Elsholtzia* genus of the family Labiatae. It is widely spread throughout the warm zone of Eurasia. It grows mainly in the north of China in Hebei, Shanxi, QTP (The Qinghai-Tibetan Plateau) and adjacent areas, including Gansu, Qinghai, and Tibet, West Sichuan, etc. (Sun et al. 1996). Although *E. densa* is considered a weed, it was also used as a medicine because of its rich nutrients and bioactive substances, especially the herb has essential oil containing bioactive compounds. 73 volatile compounds in the volatile oil have been reported (Xue et al. 2016; Zhang et al. 2019). A new study also reported the presence of 40 constituents comprising 83.3–83.7% of essential oil composition. Essential oil extracted from the leaf of *E. densa* was characterized by citral (52.2%), geranyl acetate (3.3%), and geraniol (3.1%) (Chauhan et al. 2018). So, *Elsholtzia* species are commonly used in traditional medicine to treat colds, fever, dysentery, digestion disorders, and heat stroke, among others (Liu et al. 2007). So far, the researches on the genome of *E. densa* have not been reported. Here, to develop and utilize the herbal plants better, the complete chloroplast genome sequence (Genbank accession number: MN793319) has been detected and phylogenetic analysis was also conducted, which provide more genome data information for its genetic and evolution in the family Labiatae.

In this study, the specimen *E. densa* was sampled from Qinghai lake Erlangjian scenic, Gonghe County, China (36.5785°E, 100.4911°N). A voucher specimen (FGE20197201) was stored in the Qinghai Nationality University, Qinghai, China. Total DNA was extracted from freshly collected leaf tissue using the modified CTAB method described by Rofers and Bebdich (1998). The complete chloroplast was sequenced by Illumina Hiseq 4000 (Illumina, San Diego, CA) in Genepioneer Biotechnologies Inc., Nanjing, China. The trimmed reads were mainly assembled by SPAdes (Bankevich et al. 2012), with that of its congener *Pogostemon cablin* (GenBank accession number: MF287372.1) (Zhang et al. 2019) and approximately 5.3 Gb clean data were assembled.

The 149,095 bp length circular chloroplast genome of *E. densa* was obtained, which comprised of two inverted repeats (IRs) of 25,117 bp, a large single-copy (LSC) region genome of 81,497 bp, and a small single-copy (SSC) region of 17,364 bp. The total GC content is 37.92% while the corresponding value of the LSC, SSC, and IR regions was 35.96%, 31.92%, and 43.16%, respectively. A total of 129 genes was identified, which included 84 unique protein-coding genes, 37 unique tRNA genes, and 8 unique rRNA genes.

Alignment for cpDNA *E. densa* sequences was conducted using MAFFT (https://mafft.cbrc.jp/alignment/server/). Phylogenetic relationships were evaluated based on cpDNA sequences using the maximum likelihood (ML) method. RA × ML v8.2.10 software (https://cme.h-its.org/exelixis/software.html) was used to reconstruct the phylogenetic tree with 1000 bootstraps under the GTRGAMMAI substitution model. The tree was reconstructed including *E. densa,* and 31 published complete chloroplast sequences of Labiatae and *Sesamum indicum* (Pedaliaceae) was chosen as an outgroup. Based on the phylogenetic analysis, the species from identical family were clubbed together as a monophyletic clade and all nodes showed strong support (>95%). Meanwhile, a monophyletic clade was formed including *E. densa* and *Elsholtzia splendens* (MH700782.1). A close relationship was revealed between *E. densa* and *E. splendens* (Figure 1). The results in this study will provide a useful foundation for further investigation of genetic and phylogenetic studies for the family Labiatae.

**Figure 1.**
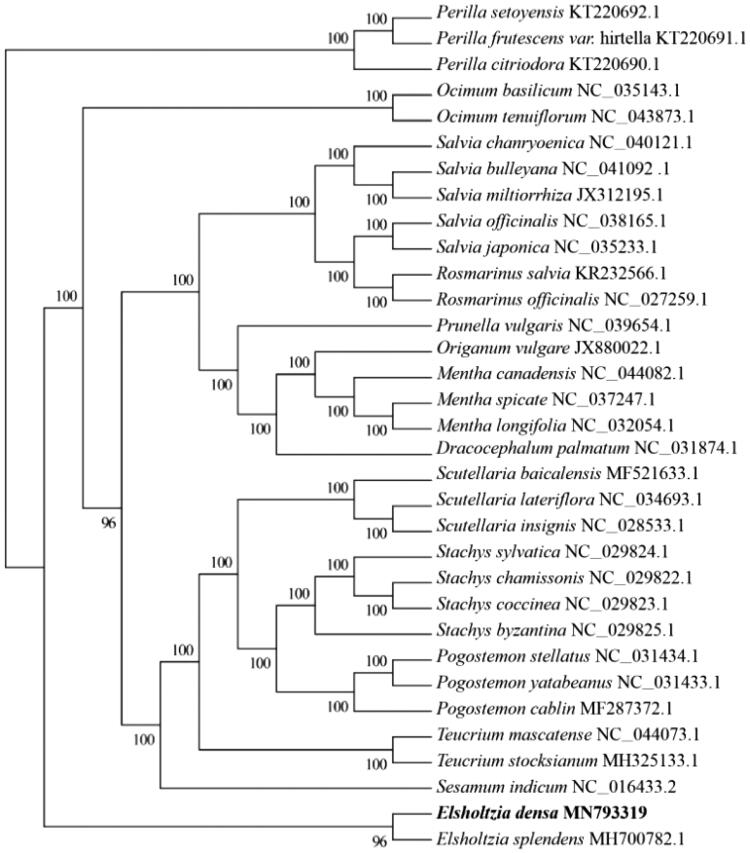
The maximum likelihood (ML) tree inferred from 31 representative chloroplast genomes of Labiatae and Pedaliaceae. The bootstrap value based on 1000 replicates is marked on each node and species accession numbers are shown in the figure.

## References

[CIT0001] Bankevich A, Nurk S, Antipov D, Gurevich AA, Dvorkin M, Kulikov AS, Lesin VM, Nikolenko SI, Pham S, Prjibelski AD, Pyshkin AV, et al. 2012. SPAdes: a new genome assembly algorithm and its applications to single-cell sequencing. J Comput Biol. 19(5):455–477. 22506599 10.1089/cmb.2012.0021 PMC3342519

[CIT0002] Chauhan A, Venkatesha KT, Padalia RC, Singh VR, Verma RS, Chandan SC. 2018. Essential oil composition of leaves and inflorescences of *Elsholtzia densa* Benth. from western Himalaya. J Essent Oil Res. 31(3):1–6.

[CIT0003] Liu A, Lee SM, Wang Y, Du G. 2007. E*lsholtzia*: review of traditional uses, chemistry and pharmacology. J Chin Pharm Sci. 16:73.

[CIT0004] Rofers SO, Bebdich AJ. 1998. Extraction of DNA from plant tissues. Plant Mol Biol. A6:1–10.

[CIT0005] Sun LP, Yin ZD, Fu ZS, Zheng SZ. 1996. The chemical constituents of *Elsholtzia densa* Benth. Acta Bot Sin. 38(8):672–676.

[CIT0006] Xue XJ, Guo ZJ, Zhang H, Liu X, Luo J, Li DD, Li J. 2016. Chemical composition, in vitro antioxidant activity and α-glucosidase inhibitory effects of the essential oil and methanolic extract of *Elsholtzia densa* Benth. Nat Prod Res. 30(23):1–5. 26784285 10.1080/14786419.2015.1135147

[CIT0007] Zhang C, Liu T, Yuan X, Huang H, Yao G, Mo X, Xue X, Yan H. 2019. The plastid genome and its implications in barcoding specific-chemotypes of the medicinal herb *Pogostemon cablin* in China. PLoS One. 14(4):e0215512. 30986249 10.1371/journal.pone.0215512 PMC6464210

[CIT0008] Zhang Z, Chen Y, Jiang X, Zhu P, Zeng Y, Tang T, Li L. 2019. Characterization of the complete chloroplast genome sequence of *Galinsoga parviflora* and its phylogenetic implications. Mitochondrial DNA Part B. 4(2):2106–2108. 33365429 10.1080/23802359.2019.1623106 PMC7687395

